# Virucidal nano-perforator of viral membrane trapping viral RNAs in the endosome

**DOI:** 10.1038/s41467-018-08138-1

**Published:** 2019-01-14

**Authors:** Byoungjae Kong, Seokoh Moon, Yuna Kim, Paul Heo, Younghun Jung, Seok-Hyeon Yu, Jinhyo Chung, Choongjin Ban, Yong Ho Kim, Paul Kim, Beom Jeung Hwang, Woo-Jae Chung, Yeon-Kyun Shin, Baik Lin Seong, Dae-Hyuk Kweon

**Affiliations:** 1 Department of Integrative Biotechnology, College of Biotechnology and Bioengineering, Sungkyunkwan University, Suwon, 16419 Republic of Korea; 2 Biomedical Institute for Convergence, Sungkyunkwan University, Suwon, 16419 Republic of Korea; 3 Department of Biotechnology, College of Life Science and Biotechnology, Yonsei University, Seoul, 03722 Republic of Korea; 4 Department of Biochemistry Biophysics and Molecular Biology, Iowa State University, Iowa, IA 50011 USA

## Abstract

Membrane-disrupting agents that selectively target virus versus host membranes could potentially inhibit a broad-spectrum of enveloped viruses, but currently such antivirals are lacking. Here, we develop a nanodisc incorporated with a decoy virus receptor that inhibits virus infection. Mechanistically, nanodiscs carrying the viral receptor sialic acid bind to influenza virions and are co-endocytosed into host cells. At low pH in the endosome, the nanodiscs rupture the viral envelope, trapping viral RNAs inside the endolysosome for enzymatic decomposition. In contrast, liposomes containing a decoy receptor show weak antiviral activity due to the lack of membrane disruption. The nanodiscs inhibit influenza virus infection and reduce morbidity and mortality in a mouse model. Our results suggest a new class of antivirals applicable to other enveloped viruses that cause irreversible physical damage specifically to virus envelope by viruses’ own fusion machine. In conclusion, the lipid nanostructure provides another dimension for antiviral activity of decoy molecules.

## Abstract (web summary)

Membrane-disrupting agents that selectively target virus versus host membranes could potentially be potent antivirals. Here the authors incorporate a decoy virus receptor into a nanodisc and show that it ruptures the viral membrane at low pH and traps viral RNAs in the endolysosome for degradation.

## Introduction

Influenza is one of the most common causes of human respiratory illnesses and shows high morbidity and mortality. Anti-influenza therapeutic options available to date are viral M2 ion channel inhibitors (e.g., amantadine and rimantadine) and neuraminidase (NA) inhibitors (e.g., oseltamivir, zanamivir and peramivir). All influenza viral proteins including hemagglutinin (HA), NA, viral nucleoprotein (NP) and viral RNA-dependent RNA polymerase are potential targets for anti-influenza drugs^1–3^. However, antivirals targeting viral proteins inevitably elicit resistance because mutants that do not respond to the drug tend to be enriched during treatment^3,4^.

In contrast, antivirals targeting the viral membrane envelope not only show broad antiviral activity but also are less likely to induce resistance because the envelope is derived from the host cell membrane which is not under direct control of the virus^5–10^. Enveloped viruses are less tolerant to chemical disinfectants such as detergents, acids and alcohols compared with naked viruses. Biologically relevant antivirals such as lysophosphotidylcholine, peptides and chemicals which affect viral membrane integrity, show broad antiviral activities^5–9^. This strong and broad-spectrum activity is due to the vital roles of the envelope in binding to receptors on the host cell membrane, transmitting the viral genome into host cells, and releasing viral progeny^10^. However, because disruption of membrane integrity is not specific to the virus, these agents are often cytotoxic, preventing their application as antivirals. Perturbing membrane integrity specifically of the viral envelope therefore is key to achieving strong, broad and less-toxic antiviral effects with lower resistance. Here, we report a virucidal nanostructure that specifically induces viruses to self-disrupt their envelope with their own fusion machinery. Antiviral nanostructures have been mostly developed as decoys. When decoy molecules that target viral proteins are contained in nanostructures of a dendrimer or a liposome, it interferes better with the virus–host interaction compared with their monomeric forms through multivalent interactions^11,12^. This report demonstrates that the plain bilayer has another dimension to its antiviral activity and can further amplify the antiviral effect of decoy molecules by inducing self-perforation of virus envelope.

## Result

### Amplification of antiviral effect by nanodisc

**Fig. 1:**
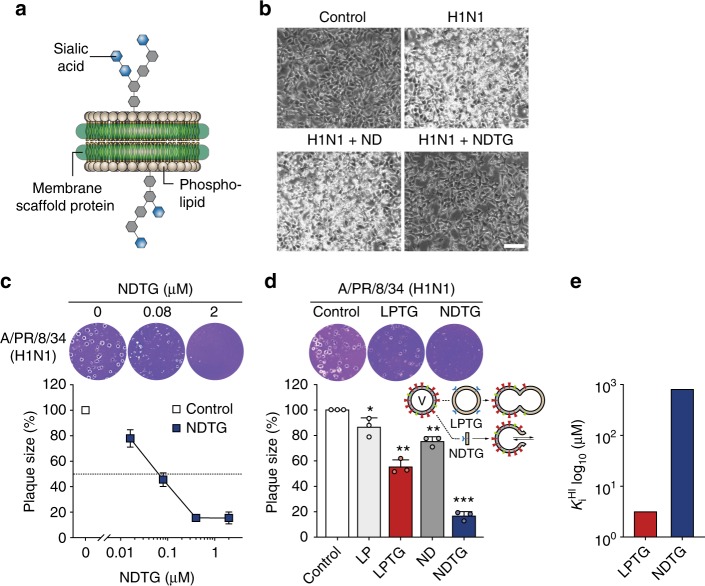
In vitro inhibitory activity of NDTG against H1N1 virus infection. **a** Cartoon representation of the total ganglioside-embedded nanodisc (NDTG). **b** Microscopy observations of virus-induced CPE reduction in MDCK cells by NDTG. MDCK cells were either uninfected (control) or challenged with A/PR/8/34 H1N1 virus (MOI = 0.1) at 25 °C for 24 h, with 0.5 μM ND or NDTG in the presence of TPCK-treated trypsin for activation of HA. CPE was observed at 24 h post-infection by inverted microscopy. Scale bar, 100 μm. **c**, **d** Evaluation of antiviral activity of NDTG against H1N1 virus by a plaque reduction assay. MDCK cells were infected with ~100 PFU of H1N1. One hour after infection, the viral inoculum was removed, and cells were overlaid with agarose containing the indicated concentrations of NDTG (**c**), or overlay supplemented with ND, NDTG, LP or LPTG at the same lipid concentration of 50 μM (**d**). Representative samples of viral plaques in each histogram are shown. Data are expressed as the mean ± standard deviation (SD) of triplicate samples. Error bars indicate SD, and asterisks indicate statistical significance determined by Student’s *t*-test (*P < 0.05; **P < 0.01; ***P < 0.001). **e** Hemagglutinin inhibition by NDTG or LPTG. The lowest lipid concentration of inhibitor that completely suppressed hemagglutination was defined as the hemagglutination inhibition constant *K*_i_^HI^

Nanodiscs are self-assembled discoidal phospholipid bilayers wrapped by two copies of amphipathic membrane scaffold protein (MSP), an engineered form of apolipoprotein A1 and a constituent of high-density lipoproteins^13^ (Fig. 1a). Nanodiscs exhibit biocompatibility and safety, enabling in vivo applications^14–16^. In contrast to liposomes, HA-mediated fusion of the viral envelope and nanodisc is predicted to form a direct passage from the environment to the inside of the viral envelope^8,17^ (Supplementary Figure 1a). Formation of a fusion pore within the nanodisc bilayer has been demonstrated by the soluble *N*-ethylmaleimide-sensitive factor attachment protein receptor (SNARE) complex^18,19^ and poliovirus^20^.

A nanodisc with affinity to influenza virus, termed as NDTG, was assembled using total ganglioside extract (TG, which is the receptor for HA binding), 1-palmitoyl-2-oleoyl-*sn*-glycero-3-phosphatidylcholine (POPC), and a membrane scaffold protein MSP1E3D1 (Fig. 1a). After assembly, NDTG was purified using size exclusion chromatography (SEC). NDTG eluted earlier than ND (i.e., nanodisc without the ganglioside receptors) (Supplementary Figure 1b). Dot-blot analysis showed that TG was successfully reconstituted into NDTG (Supplementary Figure 1c). The NDTGs showed distinct disc shape and their size distribution and three-dimensional structure were analysed using dynamic light scattering (DLS) (Supplementary Figure 1d) and negative staining electron microscopy (EM) (Supplementary Figure 1e), respectively. NDTG (14.8 nm) exhibited a slightly larger diameter than ND (13.9 nm), which was consistent with the earlier elution of NDTG in SEC. This size is big enough to engage in HA-mediated fusion which requires more than one HA trimer^21^ (Supplementary Figure 1f).

The ability of NDTG to inhibit mouse-adapted influenza virus A/PR/8/34 (H1N1) infection in vitro was assessed by cytopathic effect (CPE) reduction and plaque reduction assays. After Madin–Darby canine kidney (MDCK) cells were infected with H1N1 virus (multiplicity of infection, MOI = 0.1) at 25 °C for 1 h, the cells were treated with 0.5 µM NDTG after removing the viral inoculum. At 24 h post-infection, the cells showed a bright round shape because of CPE by viral infection (Fig. 1b). The CPE was remarkably alleviated when the cells were treated with NDTG, but not when treated with ND alone. The cells treated with NDTG at 25 °C for 1 h followed by washing and infection with the virus did not show reduced cell death. The antiviral efficacy of NDTG was examined in a plaque reduction assay, in which confluent monolayers of MDCK cells in a 6-well plate were infected with H1N1 virus (100 PFU/well) at 25 °C for 1 h to enable virus attachment. Subsequently, unbound viruses were removed, and the cells were overlaid with agar medium containing NDTG at the indicated concentrations. The size of viral plaques was quantified after 3 days (Fig. 1c). Plaque size was dramatically reduced by NDTG. The half-maximal inhibitory concentration (IC_50_) of NDTG was 72 nM for H1N1. We next compared the antiviral activity of LPTG (TG-containing liposomes) with that of NDTG. LPTG reduced plaque size by 45%, while NDTG inhibited plaque growth by 84% at the same lipid concentration (Fig. 1d). In contrast, hemagglutination inhibition by NDTG was less efficient than that by LPTG (Fig. 1e and Supplementary Figure 2). While both liposomes and nanoparticles conjugated with receptors to HA typically function as virus decoys^11,12^, the amplified antiviral activity of NDTG, compared with LPTG, indicates that the nanodisc not only functioned as a decoy, but also played a vital role in reducing virus infectivity.

### Perforation of the viral envelope by nanodisc

**Fig. 2:**
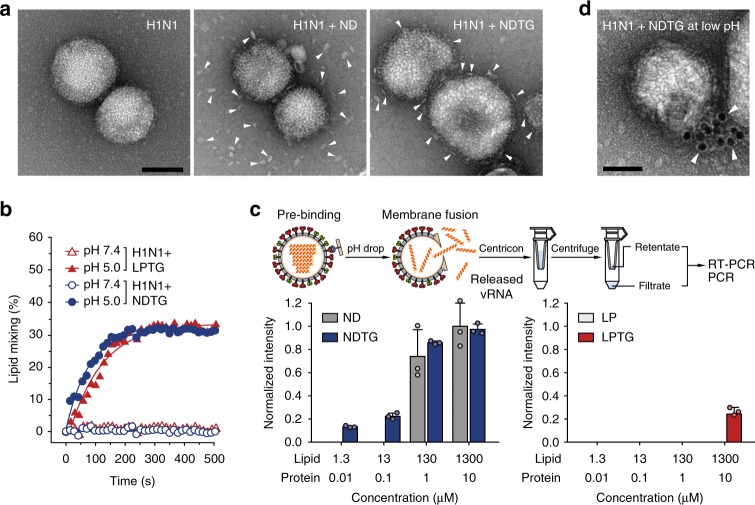
Perforation of viral envelope through membrane fusion at low pH. **a** EM analysis of H1N1 virus alone, mixture of ND and H1N1, and mixture of NDTG and H1N1. White arrows indicate ND or NDTG. Scale bar, 100 nm. **b** Lipid mixing assay monitored by dequenching of NBD fluorescence following fusion of the nanodisc and virus. **c** Release of vRNPs from viruses. After virus fusion with ND, NDTG, LP or LPTG was triggered at pH 5.0, vRNP release was analysed by RT-PCR for the viral M gene following centrifugation with a centrifugal filter. Data are expressed as the mean ± SD of three independent experiments. **d** Immunogold labelling of vRNPs released from the H1N1 virus after incubation with NDTG at low pH. White arrows indicate gold particles bound to the released vRNPs. Scale bar, 50 nm

Binding of NDTG to the influenza virus was analysed in a pull-down assay using nickel-nitrilotriacetic acid (Ni-NTA) beads (Supplementary Figure 3). Direct evidence for binding of NDTG to H1N1 virus was obtained by single-particle analysis using EM. After mixture of nanodiscs and H1N1 virus was pre-incubated at 25 °C for 1 h, small droplets of the mixture were adsorbed and negatively stained on grids. Most ND particles were found unbound, while many NDTG particles surrounded the viruses (Fig. 2a). A well-established lipid mixing assay was performed to test whether NDTG fused with viral membranes^22^. Briefly, 1,2-dipalmitoyl-*sn*-glycero-3-phosphoethanolamine-N-(7-nitro-2-1,3-benzoxadiazol-4-yl) (NBD-PE) and 1,2-dipalmitoyl-*sn*-glycero-3-phosphoethanolamine-N-(lissamine rhodamine B sulfonyl) (Rhod-PE) were added to NDTG (1.5 mol% each) as a fluorescence resonance energy transfer (FRET) pair. Upon acidification of the virus–nanodisc mixture, robust fusion of NDTG with the H1N1 virus membrane occurred with fusion levels comparable with those of LPTG (Fig. 2b). The fluorescence intensity reached a steady value within a few minutes, which agrees with the result of a previous study^23^. When assessed by static light scattering and EM, nanodiscs were stable between pH 5.0 and 7.4 (Supplementary Figure 4), consistent with a previous report^24^.

The effect of NDTG and LPTG on viral envelope integrity was compared. First, leakage of viral ribonucleoprotein complexes (vRNPs) from viruses at low pH was analysed using a centrifugal filter with a 100-kDa molecular weight cutoff (Fig. 2c and Supplementary Figure 5). When vRNPs were analysed by reverse transcription (RT)-PCR to detect the viral M gene, the second smallest of eight segments^25^, free vRNPs were detected outside the virions after the pH was lowered in the presence of NDTG (Fig. 2c). vRNPs were not released in the presence of NDTG at pH 7 or at pH 5 without NDTG (Supplementary Figure 5). In contrast, liposome–virus fusion induced minimal vRNP release from the viruses. EM followed by immunogold labelling provided additional evidence for NDTG-triggered vRNP release (Fig. 2d). After lowering the pH, in the presence of NDTG, viruses were treated with an antibody against NP, followed by treatment with secondary antibodies conjugated to 10-nm gold nanoparticles and negative staining. Gold particles were observed on the ruptured virion surface. These results suggest that the amplification of the antiviral effect by nanodiscs could be attributed to the planar nanostructure of the nanodisc, which ultimately resulted in viral envelope perforation and vRNP leakage through HA-mediated membrane fusion.

### Improvement of antiviral nano-perforator

**Fig. 3:**
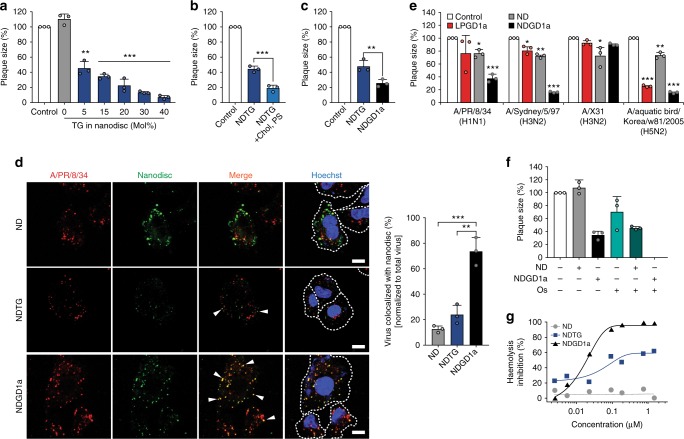
Optimisation of nano-perforator to improve anti-influenza activity. **a**–**c** Anti-influenza efficacies of nanodiscs with different formulations determined by a plaque reduction assay. Nanodiscs were prepared with various amounts of total gangliosides (**a**), with cholesterol and negatively-charged lipids (**b**), and with ganglioside GD1a (**c**). Nanodisc concentrations were 0.25 μM for (**a**) and 0.1 μM for (**b**, **c**). **d** Immunofluorescent images showing intracellular influenza virus–nanodisc colocalization in a receptor-dependent manner. A549 cells were incubated with SP-DiOC18-labelled influenza virus A/PR/8/34 (red) pre-mixed with Rhod-PE-labelled nanodiscs (green) at 37 °C and fixed at 2 h post-infection. Cell membranes and nuclei were stained with WGA-AF647 (white dashed line) and Hoechst (blue), respectively. Representative orange colocalization spots are indicated by white arrows. Histogram of virus–nanodisc colocalization is shown on the right. Scale bars, 10 μm. **e** Antiviral effects of NDGD1a (0.1 µM) against infections of multiple influenza virus strains compared with liposome containing GD1a (LPGD1a). **f** Combination effect of nanodiscs with oseltamivir (Os) on influenza virus H1N1 infectivity. Histogram of antiviral activity of co-treatment of 0.3 µM Os and 0.1 µM nanodiscs against A/PR/8/34 (H1N1). **g** Haemolysis inhibition of chicken red blood cells by nanodiscs. Data are expressed as the mean ± SD of three independent experiments (**a**–**f**). Asterisks indicate statistical significance determined by Student’s *t*-test (*P < 0.05; **P < 0.01; ***P < 0.001) (**a**–**e**)

Because the antiviral effect of NDTG was accompanied by membrane fusion, several factors potentially affecting membrane fusion were examined. First, the effect of incorporated TG receptors was determined by varying the amounts of TG in nanodiscs and performing a plaque reduction assay. Better antiviral activity was observed with higher TG content in the nanodisc (Fig. 3a). Second, the lipid composition of the nanodisc was tested. Cholesterol is known to promote hemifusion and pore widening during virus-induced membrane fusion. Additionally, negatively charged lipids, such as phosphatidylserine (PS), trigger conversion of a restricted hemifusion intermediate to the fully fused product^17,26,27^. Inclusion of these two components in NDTG significantly increased its antiviral activity (Fig. 3b). Third, the antiviral activity was improved by incorporating ganglioside GD1a receptor (Fig. 3c), which has higher affinity for influenza virus^28^.

Localisation of the virus and nanodiscs were determined by confocal microscopy (Fig. 3d). Human lung epithelial (A549) cells were incubated with mixture containing H1N1 labelled with lipophilic fluorescent dye SP-DiOC18 and nanodiscs (ND, NDTG, or NDGD1a) labelled with Rhod-PE. Imaging results indicated that NDGD1a and virus particles colocalized in the endosome. The colocalization percentage of NDGD1a and virus was significantly higher than that of NDTG, while the receptor-free ND showed very few overlapping spots. Images sectioned along *z*-axis showed that the fluorescence spots of viruses and NDGD1a were mostly present at the mid-height of the nucleus, indicating colocalization in endosomes rather than at the cell surface (Supplementary Figure 6). In contrast, orthogonal view of images showed that the receptor-free ND existed predominantly on the cell surface, while colocalized spots of NDGD1a and virus were clearly seen under the cell membrane (Supplementary Figure 6). This confirms again that NDGD1a colocalized with viruses within the cell.

Further, the antiviral activity of NDGD1a against other subtypes of influenza virus was evaluated (Fig. 3e). NDGD1a strongly inhibited the growth of influenza viruses A/Sydney/5/97 (H3N2) and A/aquatic bird/Korea/w81/2005 (H5N2) as well as of H1N1. Additionally, the nanodiscs consistently exhibited superior antiviral activity, compared with liposomes (Fig. 3e). The A/X31 (H3N2) virus was not inhibited by NDGD1a as GD1a is not a receptor for this strain^29^. Next, the combination effects with NA inhibitor oseltamivir (Os) were investigated. Plaque size was reduced by ~30 and ~65% by 3 µM Os and 0.1 µM NDGD1a, respectively. However, a combination of the two antivirals nearly completely eradicated the virus (Fig. 3f), suggesting that the membrane-targeting nanodiscs may cooperate with a protein-targeting antiviral. Finally, we employed a haemolysis inhibition assay to evaluate the inhibitory effect of NDGD1a on virus-mediated membrane fusion. When 2% chicken erythrocytes were mixed with equal volumes of NDGD1a and H1N1 in a 96-well plate, virus-induced erythrocyte lysis was decreased in a dose-dependent manner with an IC_50_ of 20 nM (Fig. 3g). This result suggests that nanodiscs containing a receptor with high affinity to the virus will interfere with fusion of the viral envelope and endosome after internalisation.

### Abortive release of vRNP in the endolysosome

**Fig. 4:**
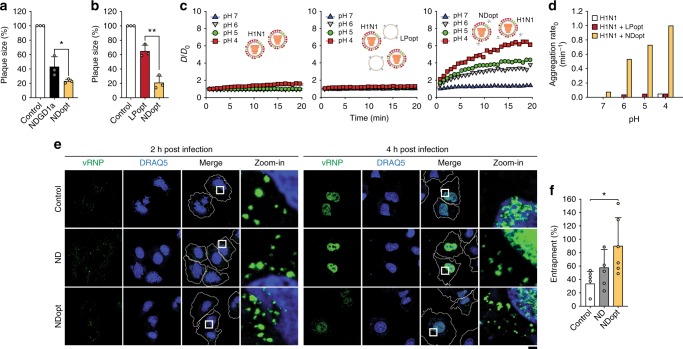
Abortive vRNP release in the endolysosome by NDopt. **a**, **b** Antiviral activity against H1N1 virus of NDopt compared with NDGD1a (50 nM) (**a**) or liposomes with the same lipid composition as NDopt (LPopt) (12.5 μM lipid) (**b**) was examined by a plaque assay (*n* = 3). **c**, **d** Aggregation of NDopt-disrupted viruses was monitored by measuring the changes in the average size of samples as a function of time. **c** Time-dependent changes in the diameter of H1N1 virus only (left panel), mixture of H1N1 and LPopt (middle panel) and mixture of H1N1 and NDopt (right panel). H1N1 virus (1 × 10^7^ PFU/mL) was mixed with PBS, LPopt (2.5 μM for lipid) or 16.6 nM NDopt. Changes in the diameter were monitored using a Zetasizer (Nano ZS90, Malvern Instruments, Ltd., Worcestershire, UK) and expressed as *D*/*D*_0_, where *D*_0_ and *D* are the averaged diameters of samples before and after vhjpH adjustment, respectively. **d** Aggregation rate_0_, calculated from the slope of *D*/*D*_0_ at the beginning of the aggregation reaction, suggests that viral envelope is efficiently disrupted by nanodiscs. **e** Influenza virus vRNP entrapment in endosome by NDopt. White squares in merge panels are enlarged to show virus entrapment (green) near the nucleus (blue) (zoom-in). Cell membranes shown as white dashed lines. Scale bars, 10 μm. **f** Quantitation of viral entrapment, which was calculated by dividing the number of vRNP spots in the endosome at 4 h post-infection by the number of vRNP spots at 2 h post-infection (*n* = 5). Data are expressed as the mean ± SD. Asterisks indicate statistical significance determined by Student’s *t*-test (*P < 0.05; **P < 0.01; ***P < 0.001) (**a**, **b**, **f**)

Because virus–erythrocyte fusion was inhibited (Fig. 3g) while virus–nanodisc fusion perforated the virus envelope (Fig. 2c), it was likely that vRNPs were not released into the cytosol but remained trapped within endolysosomes. Firstly, we combined all factors tested that improved the antiviral activity of the nanodiscs to develop NDopt. NDopt was composed of POPC, PS, cholesterol and GD1a at a molar ratio of 25:15:30:30. NDopt showed stronger antiviral activity than NDGD1a (Fig. 4a) and liposomes with the same lipid composition (LPopt) (Fig. 4b). NDopt perforated the viral envelope, leading to aggregation of viruses at low pH (Fig. 4c) and the rate of viral envelope disruption by NDopt was 20–30 times greater than that in presence of LPopt or virus alone (Fig. 4d). Additionally, the CPE was very efficiently reduced when cells were treated with a mixture of virus and NDopt (Supplementary Figure 7, Scheme A). In contrast, if the cells were treated with NDopt after virus internalisation, CPE reduction was observed only after prolonged incubation time in the presence of TPCK-treated trypsin to allow viral progeny to encounter NDopt in the medium (Supplementary Figure 7, Scheme B). These results suggest that direct contact of virus and nanodisc was indispensable for the antiviral activity of nanodiscs.

The location of vRNPs in the cell was investigated by confocal microscopy, 2 or 4 h after A549 cells were treated with H1N1 and ND or NDopt (Fig. 4e). These incubation times were predetermined because vRNPs were not transported to the nucleus at 2 h. At 4 h, numerous NP-positive nuclei were observed, with undetectable translated NPs in the cytosol (Supplementary Figure 8). Anti-NP antibodies were used to detect the location of vRNPs. Most vRNP-containing endosomes detected at 2 h post-infection disappeared because vRNPs were transported into the nucleus after 4 h of infection. In contrast, 89% of vRNP-containing endosomes found at 2 h post-infection could still be observed at 4 h in presence of NDopt (Fig. 4f), suggesting that most vRNPs of endocytosed viruses were entrapped in the endosome. Entrapment of just one viral RNA molecule of the eight inside the late endosome will not allow for virus packaging (Supplementary Note 1). Thus, NDopt evokes abortive fusion, resulting in vRNP entrapment in the endolysosome, where viral RNAs may be subjected to decomposition.

**Fig. 5:**
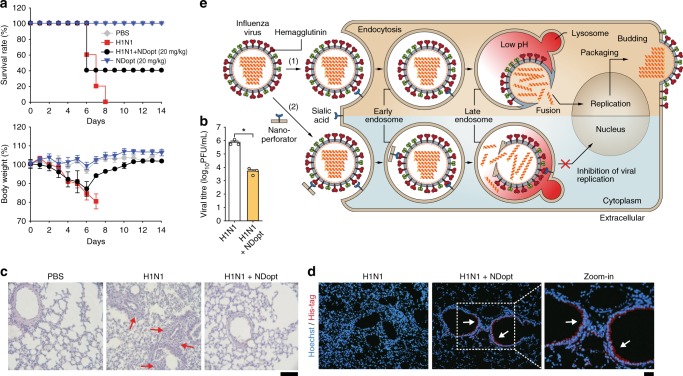
In vivo antiviral activity of NDopt. **a** Survival rates and body weight of the mice. NDopt (20 mg/kg) was intravenously administered to mice (*n* = 5/group) at 1 h after intranasal infection with mouse-lethal doses of H1N1 virus, and then once a day for 5 days. The survival rate (left) and body weight (right) of each group were monitored daily for 14 days. **b**–**d** Reduced viral replication and inflammation lesion by NDopt in the lungs of virus-infected mice. The lungs were obtained from mice administered intravenously with NDopt 5 days post-infection. **b** Reduced viral titres, as determined by a plaque assay, were observed in the lung administered with NDopt (*n* = 3). **c** Images of lung tissues stained by H&E for histopathologic examination. The representative micrographs from each group is shown at ×100 magnification. Red arrows indicate the presence of inflammatory cells in the tissue sections. Scale bars, 100 μm. **d** Representative fluorescence images of lung tissues of virus-infected mice treated with NDopt (×200 magnification). NDopt was detected using rabbit anti-His-tag antibody and Alexa Fluor 594-labelled anti-rabbit IgG antibody. White arrows indicate the presence of NDopt on the surface of the lung alveolar epithelial cells. Scale bars, 100 μm. **e** Proposed model for nano-perforator antiviral activity. Black arrows illustrate influenza virus infection pathway in the absence (1) or presence of nano-perforators (2). Data are expressed as the mean ± SD. Asterisks indicate statistical significance determined by Student’s *t*-test (*P < 0.05; **P < 0.01; ***P < 0.001) (**a**, **b**)

The in vivo efficacy of NDopt was also examined by intranasal inoculation of 6-week-old female BALB/c mice with twofold 90% lethal dose (LD_90_, 2 × 10^3^ PFU) of H1N1, followed by intravenous (IV) and intraperitoneal (IP) injection of NDopt. We used IV and IP for administration because most antivirals against influenza virus including broad neutralising antibodies are given using systemic routes^30–33^. Over the next 5 days, mice were dosed once daily with NDopt through IV, and morbidity and mortality were monitored daily by measuring body weight for 14 days post-infection (dpi). NDopt protected 40% of mice from lethality and reduced the body weight loss caused by viral infection, eventually leading to complete recovery at 14 dpi (Fig. 5a). The lungs of mice euthanized at 5 dpi were also analysed and examined for viral titre and histopathology. The lungs from NDopt-treated mice exhibited ~2-log reduction of the viral titre, compared with untreated control group (Fig. 5b). NDopt also reduced histopathological effects, such as infiltration of inflammatory cells (monocytes and neutrophils) in peribronchiolar and alveolar region of the lungs (Fig. 5c). Furthermore, we could clearly detect NDopt on the surface of lung alveoli epithelial cells of NDopt-treated mice (Fig. 5d), indicating that the in vivo antiviral effects were likely due to activity of NDopt. Additionally, when mouse received NDopt through IP, morbidity and mortality was similar to that seen after IV injection (Supplementary Figure 9a). NDopt improved the survival rates compared with LPopt, indicating that the antiviral activity augmented by its shape was conserved in vivo (Supplementary Figure 9a). Additionally, co-administration of NDopt (7.4 mg/kg) with Os (0.1 mg/kg) resulted in a higher survival rate than that seen with monotherapy (Supplementary Figure 9b).

## Discussion

The antiviral activity of decoy molecules is amplified when they are included in nanoparticles to enable multivalent interaction with target viral proteins^11,12^. In this study, we show that the structure of a planar lipid bilayer provided another dimension for antiviral activity of decoy molecules by perforating viral envelope. Antivirals that inhibit viral membrane can pose a barrier to resistance and have broad-spectrum antiviral activity^5–10^. However, most antivirals in this class are not specific to the viral membrane. We show that presence of the nanodisc led the influenza virus to self-disrupt its envelope through its own fusion machinery (Fig. 2), and also inhibited membrane fusion of endosome and viral envelope (Fig. 3g). Because of these dual functions, the nanodisc evoked abortive infection leading to entrapment of vRNPs inside the late endosome (Fig. 4e).

During membrane fusion between the viral envelope and endosomes, the initial fusion pore must be dilated further, enough for vRNPs to pass through and be released into the cytosol^34–36^ (Fig. 5e). However, the fusion pore formed within a nanodisc is <4 nm, which is likely not sufficient to allow passage of vRNPs^18,19^. Instead, it is plausible that perforation of the viral envelope at several places by the nanodisc destroyed membrane integrity, rupturing the virus. Aggregation of the virus by NDopt, but not by LPopt, supported that viral membrane integrity was efficiently disrupted by nanodisc (Fig. 4c). It is possible that the pore formed by virus–nanodisc fusion played a crucial role in reducing infectivity by providing a direct passage from the environment into the virus envelope (Supplementary Figure 1a). The nanodisc–virus fusion may induce contamination of vRNPs by endolysosomal contents and dissociation of M1 matrix proteins inside the endosome, leading to irreversible physical damage to the virus.

The viral HA trimers facilitate virus entry into the cell. The number of such HA trimers in a virion of influenza is estimated to be ~400^37^. Thus, HA inhibitors need to block a large number of HA molecules to exhibit antiviral activity. In contrast, the antiviral activity of a nano-perforator is not weakened by the large number of HA molecules, and rather takes advantage of this fact, since the probability of virus perforation is proportional to the number of HA molecules. Furthermore, nano-perforators are less likely to induce resistance, not only because they disrupt viral envelope derived from host cells but also because any potential mutations that decrease HA-mediated membrane fusion to confer resistance against the nano-perforator would also abrogate virus virulence. Because HA-mediated fusion is indispensable for all influenza viruses, perforation of the virus envelope through HA-mediated fusion may enable the development of broad-spectrum antivirals.

Nanodiscs as therapeutic agents against enveloped viruses are promising even though NDopt did not rescue 100% animals in our experiments. Importantly, nanodiscs did not cause any apparent toxicity, consistent with previous reports^14–16^. Furthermore, there are many possible approaches to improve in vivo efficacy. First, covalently circularised nanodisc (cND)^20^ may be considered because cNDs have improved stability against both heat and high-concentration-induced aggregations^38^. Second, better receptors than GD1a, including sialylneolacto-N-tetraose^12^ and the optimised glycan structure^11^, may increase the efficacy. Third, bigger and connected nanodiscs may allow for better and simultaneous perforation, respectively. Fourth, MSP of nanodisc may be modified to improve in vivo physicochemical and pharmacological properties. Fusion proteins, glycosylation and PEGylation that are widely used to improve the properties of various drugs may also work for nano-perforator. Finally, the optimal route of administration should be determined for the nano-perforator because direct administration in the respiratory tract has been shown to improve the efficacy of broad neutralising anti-influenza antibodies^39^. In conclusion, the nano-perforator suggested in this report will provoke and pave a way for further studies to develop potent therapeutic agent against enveloped viruses.

## Methods

### Cells and virus

Madin–Darby canine kidney (MDCK) cells (KCLB, 10034) were grown in minimum essential medium supplemented with 10% foetal bovine serum, 100 U/mL of penicillin G, 100 µg/mL of streptomycin and 0.25 µg/mL amphotericin B. Human epithelial lung carcinoma A549 cells (KCLB, 10185) were cultivated in Roswell Park Memorial Institute 1640 (RPMI) medium containing the same supplements as MDCK cell medium. The cell lines were purchased from the Korean Cell Line Bank and verified negative for mycoplasma. The cells were grown as monolayers at 37 °C in a 5% CO_2_ incubator and passaged biweekly. All media and reagents used for cell cultures were obtained from Hyclone (Logan, UT, USA). The influenza strains A/Puerto Rico/8/34 (H1N1), A/Sydney/5/97 (H3N2), A/X31 (H3N2) and A/aquatic bird/Korea/w81/2005 (H5N2) were propagated in the allantoic cavities of 10-day-old embryonated eggs at 37 °C and 40% humidity. After 3 days, the egg fluid was harvested and cleared by a four-step discontinuous sucrose gradient [each 8 mL of 50, 40, 30 and 20% (w/v) sucrose in phosphate-buffered saline (PBS) (137 mM NaCl, 10 mM Na_2_HPO_4_, 2.7 mM KCl, 1.8 mM KH_2_PO_4_ and pH 7.4)]. Viral titres were determined in MDCK cells by plaque assay.

### MSP expression and purification

The plasmid construct used in this study was the MSP1E3D1 expression vector (pMSP1E3D1), which was purchased from Addgene, Inc. (Cambridge, MA, USA). MSPs were expressed using lipopolysaccharide-free *Escherichia coli* (ClearColi^TM^ BL21 (DE3)) (60810, Lucigen, Middleton, WI, USA) and purified as follows. Luria-Bertani (LB) medium of 10  mL (244610, BD Biosciences, San Diego, CA, USA) containing 50 µg/mL kanamycin (KB0286, BIO BASIC, Inc., Markham, ON, Canada) were inoculated (0.1% (v/v)) with a cell culture stock from a single colony and the culture was grown at 37 °C with shaking (250 rpm) overnight. Next, 600 mL of LB medium supplemented with 50 µg/mL kanamycin was inoculated with 1% (v/v) overnight-grown culture and incubated at 37 °C and 150 rpm. When the OD600 reached 0.5–0.8 (~2–3 h), 1 mM of isopropyl β-d-thiogalactoside (12481C100, Gold Biotechnology, Inc., St. Louis, MO, USA) was added for induction. After 1 h, the temperature and shaking speed were lowered to 28 °C and 120 rpm, respectively, followed by further growth for 4 h. The cells were harvested by centrifugation at 7000 × *g* for 10 min, resuspended in 10 mL of chilled basic buffer (40 mM Tris/HCl, 0.3 M NaCl, pH 8.0), and lysed by sonication (50% amplitude, 1.5 min net sonication, 1 s ON – 1 s OFF) in the presence of 1 mM AEBSF (ALX-270-022-G001, Enzo Life Sciences, Inc., Farmingdale, NY, USA) and 0.1% (v/v) Triton X-100. The lysate was clarified by centrifugation (18,000 × *g* for 60 min) and bound to Ni-NTA agarose beads (30230, Qiagen, Hilden, Germany) equilibrated with basic buffer for 2 h at 4 °C with rotation. Next, the beads were washed with 5 column volumes of each of the following: (1) basic buffer + 0.1% Triton X-100, pH 8.0, (2) basic buffer + 50 mM sodium cholate, pH 8.0, (3) basic buffer and (4) basic buffer + 20 mM imidazole, pH 8.0. MSPs were eluted with elution buffer (basic buffer + 500 mM imidazole, pH 7.4). Excess imidazole was removed using a gel-filtration column (PD-10 desalting column) (17-0851-01, GE Healthcare, Little Chalfont, UK). To obtain highly pure MSPs with low endotoxin levels, 5 M urea was added to the MSP elute to a final urea concentration of 2 M^40^. The mixture was incubated at 4 °C for 6 h in a vertical rotator. After washing out unbound proteins with 4 column volumes of the 2 M urea buffer, the MSP was eluted from the column by increasing the salt concentration, starting with 150 mM NaCl. The buffer was exchanged with basic buffer using an Amicon Ultra (0.5 mL, 10 kDa cutoff) centrifugal filter (UFC5010BK, EMD Millipore, Billerica, MA, USA). The MSPs were stored at –80 °C with 10% (v/v) glycerol for long-term storage. Protein concentration was determined using the DC protein assay (5000111, Bio-Rad, Hercules, CA, USA) with bovine serum albumin (BSA) as the standard, and protein purity was confirmed by SDS-PAGE.

### Lipids and gangliosides

The lipids 1-palmitoyl-2-oleoyl-*sn*-glycero-3-phosphatidylcholine (POPC) (#850457), 1,2-dioleoyl-*sn*-glycero-3-phospho-l-serine (DOPS) (sodium salt, #840035), cholesterol (#700000), 1,2-dipalmitoyl-*sn*-glycero-3-phosphoethanolamine-*N*-(7-nitro-2-1,3-benzoxadiazol-4-yl) (NBD-PE) (ammonium salt, #810144), 1,2-dipalmitoyl-*sn*-glycero-3-phosphoethanolamine-*N*-(lissamine rhodamine B sulfonyl) (Rhod-PE) (ammonium salt, #810158), and total ganglioside extract (TG) (ammonium salt, #860053) were purchased from Avanti Polar Lipids (Alabaster, AL, USA). Ganglioside GD1a (disodium salt, ALX-302-007-M005) was obtained from Enzo Life Sciences.

### Nanodisc preparation

Lipid mixtures prepared at the desired molar ratio were treated with nitrogen gas to evaporate the solvents. Typically, the molar ratio of phospholipids and gangliosides was 100:0 for empty discs or 80:20 for ganglioside-embedded nanodiscs, and the lipid composition was adjusted as needed. The resulting lipid film was placed in a vacuum overnight (at least 2 h) and hydrated with nanodisc assembly buffer (10 mM Tris/HCl, 100 mM NaCl, 0.5 mM EDTA, pH 7.4) supplemented with 50 mM sodium cholate by vortexing vigorously at 25 °C for 5 min. The lipid–detergent solution was sonicated at 55 °C for 15 min, and then placed on ice for 10 min. MSP stock solution was added to chilled lipid–detergent solution (lipid to protein ratio was 125:1 for MSP1E3D1^41^) and vortexed every 20 min for 2 h. SM-2 bio-beads (#1523920, Bio-Rad) were added to the lipid–detergent/MSP mixture, followed by overnight agitation at 4 °C to remove the detergent. Nanodisc preparations were purified by SEC using a Superdex 200 10/300 GL column (GE Healthcare) equilibrated with PBS (pH 7.4). Fractions corresponding to the size of each nanodisc were collected and concentrated using an Amicon Ultra (0.5 mL, 10 kDa cutoff) centrifugal filter. The nanodiscs were analysed by SDS-PAGE and western blotting to quantify MSPs and gangliosides, respectively, and stored at –80 °C with 10% glycerol until use. ImageJ software (NIH, Bethesda, MD, USA) was used for quantification of gel images.

### Liposome preparation

A lipid film for liposomes was prepared in the desired ratio in the same manner as was done for nanodisc preparation. After overnight vacuum treatment, an aqueous lipid solution was obtained by dissolving the lipid film in PBS and vortexing for at least 5 min. Lipid solution was subjected to 5 freeze–thaw cycles by placing the sample in liquid nitrogen and 42 °C water, followed by an extrusion step with 21 passes through a polycarbonate membrane of 100-nm pores (#610005, Avanti Polar Lipids) using two clean gas-tight syringes (250 µL) (#610015, Avanti Polar Lipids). Extruded liposome samples were stored at 4 °C until use.

### Dot-blot analysis

Nanodisc preparations or virus elution samples were spotted on a nitrocellulose blotting membrane (GE Healthcare). The blotted membranes were dried at 25 °C for 1 h and washed with TBST (10 mM Tris/HCl, 150 mM NaCl, 0.05% (v/v) Tween-20, pH 8.0) three times. After blocking in TBST containing 0.5% BSA and 5% (w/v) skim milk for 1 h at 25 °C, mouse anti-GT1b ganglioside monoclonal antibody (1:1000 dilution) (MAB5608, EMD Millipore) or anti-influenza A H1N1 HA monoclonal antibody (1:2000 dilution) (5315-2907, Bio-Rad) in TBST supplemented with 1% BSA and 0.01% (w/v) sodium azide was added and incubated at 4 °C overnight with constant shaking. The membranes were washed thrice, for 10 min each time, with TBST, and then incubated with horseradish peroxidase-conjugated goat anti-mouse IgG secondary antibodies (1:5000 dilution) (A4416, Sigma-Aldrich, St. Louis, MO, USA) dissolved in blocking solution for 1 h at 25 °C. The membranes were again washed thrice, for 10 min each time, with TBST and visualised by chemiluminescence with WESTSAVE Gold (LF-QC0103, Young In Frontier Co., Seoul, Korea).

### Light scattering

The hydrodynamic diameter and distributions of nanodiscs and liposomes were measured using a DynaPro NanoStar DLS instrument (Wyatt Technologies, Goleta, CA, USA). Each measurement was carried out 10 times per sample at 25 °C, followed by analysis using the instrument software (Dynamics version 7.0). Samples were prepared in PBS at a final concentration of 5 µM for analysis. The pH sensitivity of nanodiscs and liposomes, prepared in PBS at a final lipid concentration of 1.25 mM, was monitored as a function of time by right-angle light scattering using a SpectraMax M2 spectrophotometer (Molecular Devices, Sunnyvale, CA, USA) with a quartz cell containing a 10-mm path length set in a cell holder at 25 °C. At the zero-time point, the samples were treated with citric acid to lower the pH or 1% Triton X-100 to control structural destruction. Excitation and emission wavelengths were 500 nm.

### Electron microscopy and Immunogold labelling

The negative staining procedure was slightly modified from previously described protocols^42^. Small droplets (10 µL) of samples were placed on carbon-coated nickel grids (TED PELLA, Inc., Redding, CA, USA) for 3 min, followed by removal of excess sample by blotting with a filter paper. Next, the grids were washed twice with water droplets, and then uranyl acetate (UA) was used to stain the droplets again. After treating the grids with UA-stained droplets for 1 min, excess stain was blotted and the grids were air-dried until TEM analysis. For immunoelectron microscopy, a mixture of viruses and nanodiscs was preincubated at 25 °C for 20 min, and then the pH was adjusted to 5.0 followed by incubation for 10 min. These steps were conducted on the grids. Next, the grids were washed with PBS for 2 min, blocked with 1% (w/v) BSA solution for 30 min, and treated with mouse anti-NP monoclonal antibody (1:100 dilution) (ab20343, Abcam, Cambridge, UK) dissolved in PBS supplemented with 1% BSA for 1 h at 25 °C. After washing the grid with 1% BSA solution for 1 min and PBS for another 3 min, goat anti-mouse antibody conjugated with 10-nm gold (1:50 dilution) (ab39619, Abcam) was added to the grid for 45 min at 25 °C. After washing with PBS three times, the samples were fixed with 2% (v/v) formaldehyde and negatively stained as described above. All specimens were analysed by energy-filtering TEM using a Libra 120 (Carl Zeiss, Oberkochen, Germany), which was operated at an accelerating voltage of 120 kV.

### Cytopathic effect (CPE) reduction assay

MDCK cells in 12-well plates were first infected with influenza A/Puerto Rico/8/34 (H1N1) (MOI = 0.1) for 1 h at 25 °C, and unbound viruses were removed with PBS. Subsequently, the cells were treated with nanodiscs at a final concentration of 0.5 µM, and then CPE was evaluated at 24 h post-infection using an inverted Leica DMi8 microscope with a 10 × objective lens (Leica Microsystems, Wetzlar, Germany).

For quantitative CPE reduction assay, 20,000 cells/200 µL MDCK cells were seeded into a well of a 96-well plate and incubated for 24 h. (Scheme A) H1N1 was diluted to MOI = 1.0 and pre-mixed with nanodiscs at 37 °C for 1 h before infection. Nanodiscs were serially diluted from 5 µM by fivefold. MDCK cells then were infected with H1N1–nanodisc mixtures at 37 °C for 1 h. Unbound viruses were removed with PBS washing. Subsequently, the cells were treated with MEM containing 10% foetal bovine serum, 100 U/mL of penicillin G, 100 µg/mL of streptomycin and 0.25 µg/mL amphotericin B, and then incubated in 5% CO_2_ at 37 °C for 16 h. (Scheme B) Seeded MDCK cells were infected with H1N1 (MOI = 0.1) at 37 °C for 1 h followed by washing with PBS. The cells were then treated with MEM containing 10% FBS, antibiotics, 2 μg/ml TPCK-treated trypsin and nanodiscs (fivefold diluted from 5 µM). MDCK cells were incubated in 5% CO_2_ at 37 °C for 48 h for multiple infection. For both scheme A and B, CPE was evaluated after fixation with 4% formaldehyde and stained with 0.5% (w/v) crystal violet. Crystal violet was dissolved to methanol and absorbance at 570 nm was measured by spectrophotometer.

### Plaque reduction assay

The antiviral effect of ganglioside-embedded nanodiscs was mainly evaluated by a plaque reduction assay. Confluent MDCK cells seeded into 6-well plates were washed twice with PBS, inoculated with the mouse-adapted influenza strain A/Puerto Rico/8/34 H1N1 (100 PFU/well), and incubated at 25 °C for 1 h with gentle agitation every 15 min. After two washes with PBS, the cells were treated with overlay consisting of 1% (w/v) agarose, 1 × Dulbecco’s modified eagle medium (Hyclone), 2 µg/mL tosyl phenylalanyl chloromethyl ketone (TPCK)-treated trypsin for HA activation, and each inhibitor at different concentrations. The plates were incubated at 37 °C in 5% CO_2_ for 3 days, followed by fixation of the cell monolayer with 4% formaldehyde and staining with 0.5% (w/v) crystal violet for visualisation. The size of plaques was counted and measured using ImageJ software.

### Hemagglutination inhibition (HI) assay

The ability of nanodiscs to inhibit hemagglutination was examined using a standard HI assay protocol^43^. HA titres of the virus were determined immediately before the hemagglutination inhibition study. The nanodiscs (25 µL) and liposomes (25 µL) were serially diluted two-fold with PBS, and then mixed with 25 µL four-HA units of A/PR/8/34 H1N1 virus in a U-type 96-well plate at 4 °C for 1 h. Subsequently, 50 µL of 1% (w/v) chicken red blood cells (cRBCs) was added to the mixture, followed by incubation for 60 min at 25 °C. The maximum dilution of the agents showing complete inhibition of hemagglutination was defined as the hemagglutination inhibition constant *K*_i_^HI^.

### Pull-down assay

A pull-down assay was performed to measure the binding of influenza viruses to nanodiscs. Ni-NTA agarose beads that had been prewashed with washing buffer (PBS supplemented with 5 mM imidazole, pH 7.4; this buffer was used at all washing steps of the assay) were incubated with nanodiscs at 4 °C for 2 h with rotation. Following another washing step, ND/NDTG–bead complexes were treated with viruses (~1 × 10^8^ PFU) at 4 °C for 2 h with rotation. The viruses bound to beads were eluted with elution buffer (PBS supplemented with 500 mM imidazole, pH 7.4) after sufficient washing to remove unbound viruses. Excess imidazole in the elute was removed with an Amicon Ultra (0.5 mL, 10 kDa cutoff) centrifugal filter, followed by dot-blot analysis to detect the viruses as described above.

### Lipid-mixing assay

Virus–nanodisc fusion was carried out to investigate the membrane fusion of viruses and nanodiscs/liposomes. Nanodiscs and liposomes were labelled with NBD-PE and Rhod-PE (1.5 mol% each), and virus fusion was monitored by dequenching of NBD fluorescence (Ex 465/Em 530) using a Spectramax M2 spectrophotometer. After activation of HA of the virus using 5 μg/mL of TPCK-treated trypsin at 37 °C for 10 min, the virus (50 μg total viral protein) was mixed with target nanodiscs or liposomes (70 μM total lipid) in a 384-well white plate, and then the temperature of the mixture was equilibrated at 37 °C for 30 min. Fusion was triggered by acidification to pH 5.0 using 100 mM citric acid stock solution. After 1 h, Triton X-100 was added to the mixture to a final concentration of 0.1% to obtain the maximum NBD signal. Lipid mixing values were collected and normalised as follows: *F* = (*F*_obs_ – *F*_0_)/(*F*_max_ – *F*_0_), where *F*_0_ is the fluorescence intensity before acidification and *F*_max_ is the fluorescence value after adding Triton X-100.

### vRNP release assay

This assay was performed to test whether vRNPs are released by membrane fusion with nanodiscs. The viruses, nanodiscs and liposomes used in this assay were prepared as in the lipid mixing assay, except that fluorescent labels were omitted. Viruses (10 µM viral lipid) mixed with nanodiscs or liposomes at different concentrations were incubated at 37 °C for 15 min with constant shaking, which allowed for nanodisc/liposome binding to virus. Fusion was induced by lowering the pH to 5.0 using 100 mM citric acid, followed by further incubation at 37 °C for 20 min. To selectively separate the released viral RNPs, the reaction mixture was added to an Amicon Ultra (0.5 mL, 100-kDa cutoff) centrifugal filter and then centrifuged at 12,000 × *g* for 10 min. The volumes of both the resultant filtrate and retentate were adjusted to be equal to the total reaction volume. To confirm the release of vRNPs, reverse transcription (RT)-PCR was carried out using M-MLV reverse transcriptase (M1705, Promega, Madison, WI, USA) according to the manufacturer’s protocol using primers for the viral M gene of A/Puerto Rico/8/34; 5′-TGCACTTTGACATTGTGGATTCTTG-3′ (M_FW) and 5′-CCCTCATAGACTTTGGCACTCC-3′ (M_BW)^44^. The coding region of the M sequence in the RT product was amplified with the described primers by using rTaq Plus 5 × PCR Master Mix (ELPIS-BIOTECH, Inc., Daejeon, Korea) according to the manufacturer’s protocol under the following thermal cycling conditions: 30 cycles of 95 °C for 10 s, 56 °C for 10 s, and 72 °C for 10 s. PCR products were separated by electrophoresis at 135 V for 20 min on a 1% agarose (Cosmogenetech, Inc., Korea) gel and visualised with a UV transilluminator. To quantify the released vRNPs, gel bands were analysed with ImageJ software.

### Immunofluorescence staining

For colocalization analysis, influenza A/Puerto Rico/8/34 (H1N1) and nanodiscs were labelled with the lipophilic fluorescent dyes SP-DiOC18 (D7778, Invitrogen, Carlsbad, CA, USA) and Rhod-PE, respectively. The labelled viruses and nanodiscs were premixed for 2 h, and then incubated with A549 cells at 37 °C. After incubation, the cells were fixed for 15 min with 3.7% formaldehyde in PBS and stained with Hoechst 33258 (Invitrogen). The images were taken under a Carl Zeiss LSM 700 confocal microscope with a 40 × objective (C Apo 40 × /1.2 W DICIII, FWD = 0.28 mm, Carl Zeiss) and captured with Zen Software (Zeiss). For the entrapment study, A549 cells were treated with H1N1 virus in the presence of nanodiscs on ice for 1 h. The cells were washed with PBS, treated with RPMI medium, and incubated at 37 °C. At 2 or 4 h post-infection, the cells were fixed for 15 min with 3.7% paraformaldehyde in PBS and permeabilised for 5 min with 0.5% Triton X-100 in PBS. After blocking with 3% BSA in PBS, the cells were incubated with primary mouse anti-NP monoclonal antibody (1:260 dilution) (ab20343, Abcam) at 25 °C for 1 h. After washing with PBS, the slides were incubated with Alexa Fluor 488-conjugated goat anti-mouse antibody (1:200 dilution) (A-11029, Invitrogen) at 25 °C for 1 h, washed with PBS, and mounted with ProLongTM Gold Antifade Mountant (P10144, Molecular Probes, Eugene, OR, USA). Nuclei were stained with DRAQ5 (62252, Invitrogen). Images were acquired using a Leica TCS SP8 HyVolution confocal microscope with a ×40 objective (HC PL APO 40 × /1.10 W CORR CS2, FWD = 0.65 mm, Leica). All immunofluorescence images were acquired at 25 °C. Images presented together were processed identically.

### Haemolysis inhibition assay

The haemolysis inhibition assay for measuring membrane fusion between virus and cRBCs was conducted as follows. 100 µL of influenza virus (1 × 10^6^ PFU/mL) was premixed with 100 µL of serially diluted nanodiscs for 1 h at 37 °C in a 96-well plate. Next, 100 µL of 2% cRBCs were incubated with a mixture of viruses and nanodiscs for 30 min at the same temperature with continuous shaking, followed by acidification with 0.5 M sodium citrate (pH 5.2). After 1 h, 200 µL of the supernatant was collected by centrifugation at 400 × *g* for 8 min and used to measure the absorbance at 540 nm with a SpectraMax M2 spectrophotometer.

### Animals and evaluation of antiviral activity in vivo

Six-week-old female Balb/c mice (average weight, 17.0 ± 2.0 g) (Koatech, Inc., Korea) were housed at five per cage under a 12-h light–dark cycle with access to food and water ad libitum. Nanodiscs were diluted with sterilised PBS for a final concentration before use. The mice were infected intranasally with 50 µL of two-fold 90% lethal doses (LD_90_) of A/PR/8/34 H1N1 virus (2 × 10^3^ PFU). Mice (five in each group) were intravenously administered with NDopt (20 mg/kg) at 1 h after virus challenge, and then once a day for 5 days beginning on the day of viral challenge. Five mice in the negative control group were administered PBS. Body weight was measured daily and survival was evaluated. The lung tissues of mice from each group were collected at Day 3, weighed, and homogenised in 1 × PBS for determination of viral titres using plaque assay. For the co-administration study, serially diluted oseltamivir phosphate (SML1606, Sigma-Aldrich) was administered to five mice in each group (0.1, 1, and 10 mg/kg oseltamivir phosphate) twice per day as described above and the appropriate doses of oseltamivir phosphate were estimated for co-administration with NDopt. One hour after viral challenge, the mice (five in each group) were intraperitoneally administered with 0.1 mg/kg oseltamivir phosphate and 7.4 mg/kg NDopt twice per day for 5 days beginning on the day of viral infection, and the synergetic antiviral effect was evaluated and compared with the monotherapy (0.1 mg/kg of oseltamivir phosphate or 7.4 mg/kg NDopt) and negative control (PBS) groups. When a mouse lost more than 25% of its initial body weight, it was defined as dead and humanely killed. The remaining mice were killed at the end of the experiment at 14 days post-infection. Randomisation was not conducted to allocate animals to experimental groups and the animal studies were not blinded. All animal experiments complied with the policies of the Institutional Animal Care and Use Committee, International Vaccine Institute (IACUC number: IACUC PN 2017-013).

### Histopathological staining

Lung tissues collected from the mice euthanized on Day 5 in each group were weighed and immediately fixed in 10% buffered formalin, dehydrated and embedded in paraffin wax. Sections of 5 μm thickness were mounted on slides. Histopathological changes were examined by hematoxylin and eosin (H&E) staining using a light microscope.

### Immunohistology

For immunofluorescent analysis, tissue sections (5 μm thickness) were washed in PBS and then blocked for 1 h with PBS containing 10% foetal bovine serum and 0.2% Triton X-100. Tissue sections were stained for a histidine tag (His-tag) of MSP1E3D1 using polyclonal rabbit His-tag antibody (1:200 dilution) (710286, Invitrogen) followed by goat anti-rabbit IgG Alexa Fluor 594 secondary antibody (1:200 dilution) (A-11012, Invitrogen). Samples were further stained with Hoechst 33258 (Invitrogen) at 25 °C for 5 min. Slide sections were mounted in ProLongTM Gold Antifade Mountant (P10144, Molecular Probes) before images from tissue samples were acquired using a Zeiss LSM 700 confocal microscope with 200x magnitude.

### Statistics

No statistical methods were used to predetermine the sample size. The experiments were not randomised and investigators were not blinded to allocation during experiments and outcome assessments. Each experiment was repeated at least three times. All statistical analysis was performed using SPSS 18.0 software (SPSS, Inc., Chicago, IL, USA). Statistical significance was determined by Student’s *t*-test for comparisons. Values of *p* < 0.05 were considered significant.

## Supplementary information

Supplementary Information

## Data Availability

The data that support the findings of this study are available from the corresponding author upon reasonable request.
