# Supplementary material for: Virucidal nano-perforator of viral membrane trapping viral RNAs in the endosome
Source: Nat Commun. 2019 Jan 14;10:185. doi: 10.1038/s41467-018-08138-1 (PMC6331592; doi:10.1038/s41467-018-08138-1)
Supplement: Supplementary file 1 — Supplementary Information [file 41467_2018_8138_MOESM1_ESM.pdf]

Supplementary information

Virucidal nano-perforator of viral membrane trapping viral RNAs in the endosome

Byoungjae Kong, Seokoh Moon et al.

Contents

Supplementary Figures 1-9

Supplementary Note

Supplementary references

Supplementary Figures

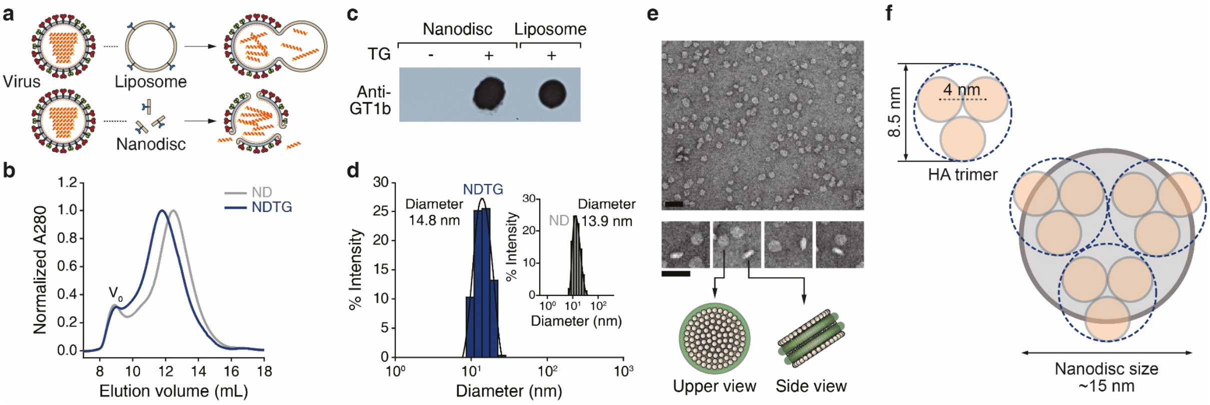

Supplementary Figure 1 | Preparation and characterization of nanodiscs with total ganglioside extract (TG). a, Comparison of fusion pore facet after fusion of viral envelope with nanodisc or liposome. **b**, SEC elution profiles of nanodiscs. Elution volume of NDTG and receptor-free ND were 11.8 and 12.5 mL, respectively. The nanodiscs eluted between 11–14 mL were used for further studies. V_o, void volume. **c**, Dot-blot analysis of NDTG showing reconstitution of TG into nanodisc, detected by anti-GT1b antibody. LPTG was used as a positive control. **d**, DLS histograms of nanodiscs. The average hydrodynamic diameters of ND (inset) and NDTG were estimated as 13.9 and 14.8 nm, respectively. **e**, EM images of negatively stained nanodiscs viewed perpendicularly to the bilayer and in the plane of the bilayer, which is illustrated at the bottom of the drawings. Scale bar, 50 nm. **f**, The size of nanodisc is big enough to interact with a few HA trimers on the virus surface. It is thought that a single HA trimer is not sufficient for fusion because the HA molecule that binds the target-membrane cannot engage in fusion[1,2,3]. The illustration shows that nanodisc with a diameter ~15 nm is big enough to interact with a few HA trimers on the virus surface.

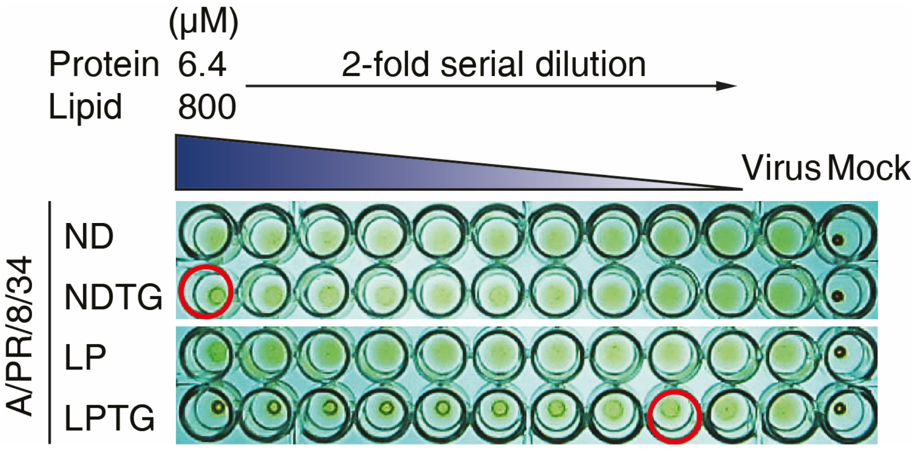

Supplementary Figure 2 | Inhibitory effect of liposome against hemagglutination is stronger than nanodisc. Hemagglutination inhibition assay was performed using chicken red blood cells (cRBCs). A sample of 25 μL of nanodiscs (ND/NDTG) or liposomes (LP/LPTG) from a two-fold serial dilution with PBS was added to 25 μL of four-fold the HA units (4 HAU) of influenza strain A/PR/8/34, followed by addition of 50 μL of 1% (w/v) cRBC to each well. After incubation at 25°C for 60 min, the maximum dilution of samples showing complete inhibition of hemagglutination was defined as the titre of hemagglutination inhibition (red circle). PBS without virus (mock) was used as a positive control, while virus alone (virus) was used as a negative control.

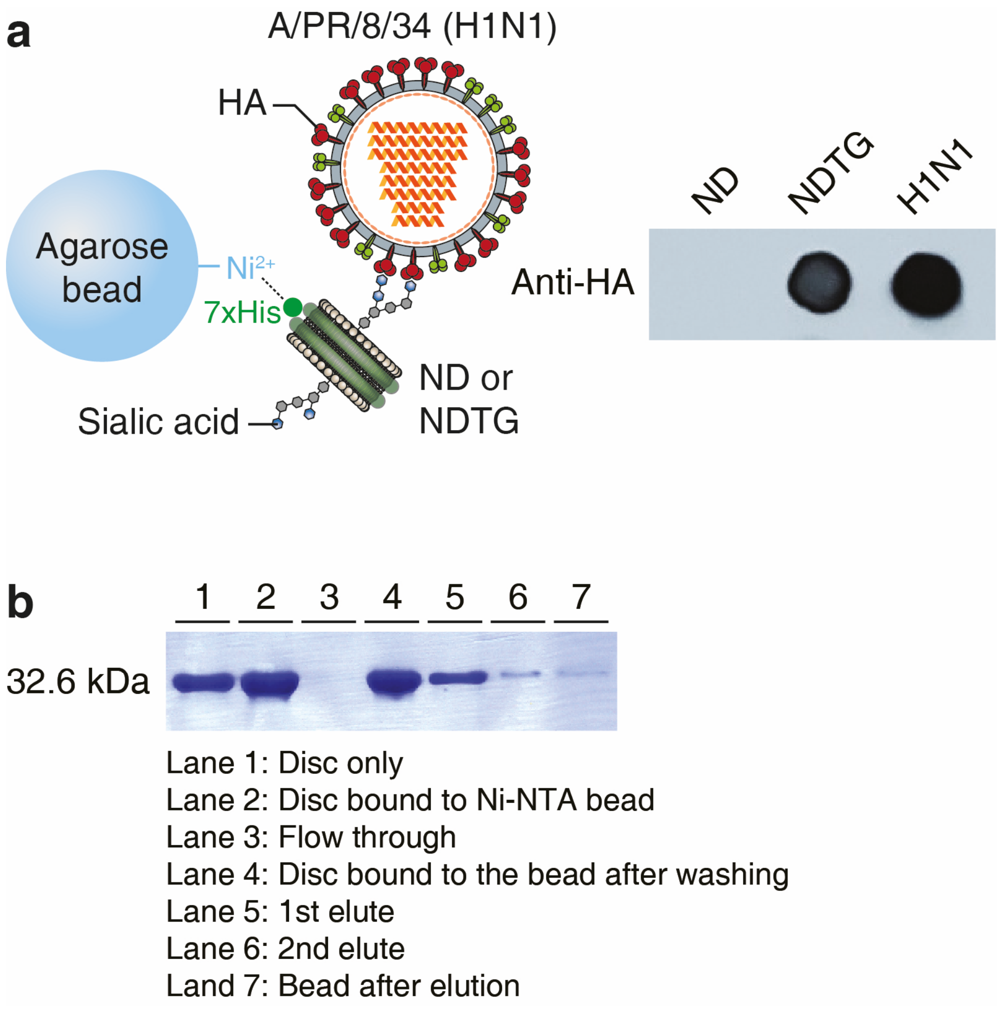

Supplementary Figure 3 | Binding of virus to nanodiscs on nickel agarose beads. a, Binding of influenza viruses to NDTG. A dot-blot assay with an anti-HA antibody was performed to detect the virus. H1N1 virus was used as a positive control. **b**, Nanodiscs (50 µg protein) were pre-bound to Ni-NTA beads equilibrated with PBS (three times) via a histidine tag (His-tag) at the N-terminus of MSP1E3D1 (32.6 kDa). After binding at 4°C for 2 h, the nanodisc–bead complex was washed with PBS and then eluted with elution buffer. Sampling was performed at each step and a description of each lane is indicated.

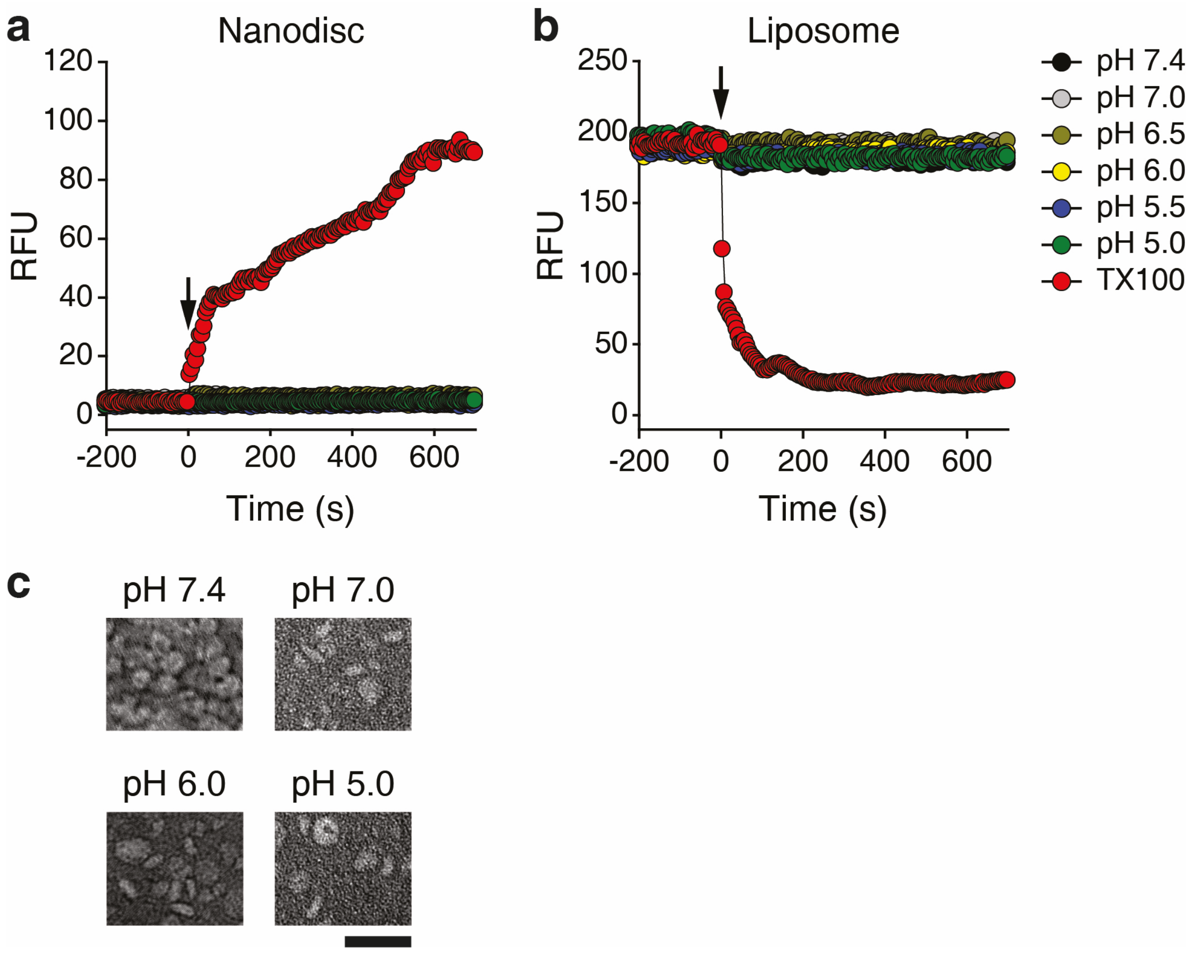

Supplementary Figure 4 | Stability test of nanodiscs. a–c, Nanodiscs are resistant against pH changes between pH 5.0 and 7.4, as confirmed by right-angle light scattering (**a** and **b**) and EM analysis (**c**). **a**, Light scattering analysis of 7.5 μM nanodiscs at various pH levels. pH was adjusted at the 0 s time point (black arrow) with 0.1 M citric acid, and liposome (2 mM lipids) was used as a control (**b**). Triton X-100 (0.1% (v/v)) was used to disrupt the membrane structure of nanodiscs and liposomes. Nanodiscs and liposomes were stable in the pH range of 5.0–7.4. **c**, EM images of nanodiscs at various pH levels. pH of nanodisc sample was adjusted with 0.1 M citric acid as indicated. After incubation for 1 h at 25°C, the samples were applied to a carbon-coated nickel grid and then negatively stained with 2% uranyl acetate. Scale bar, 50 nm.

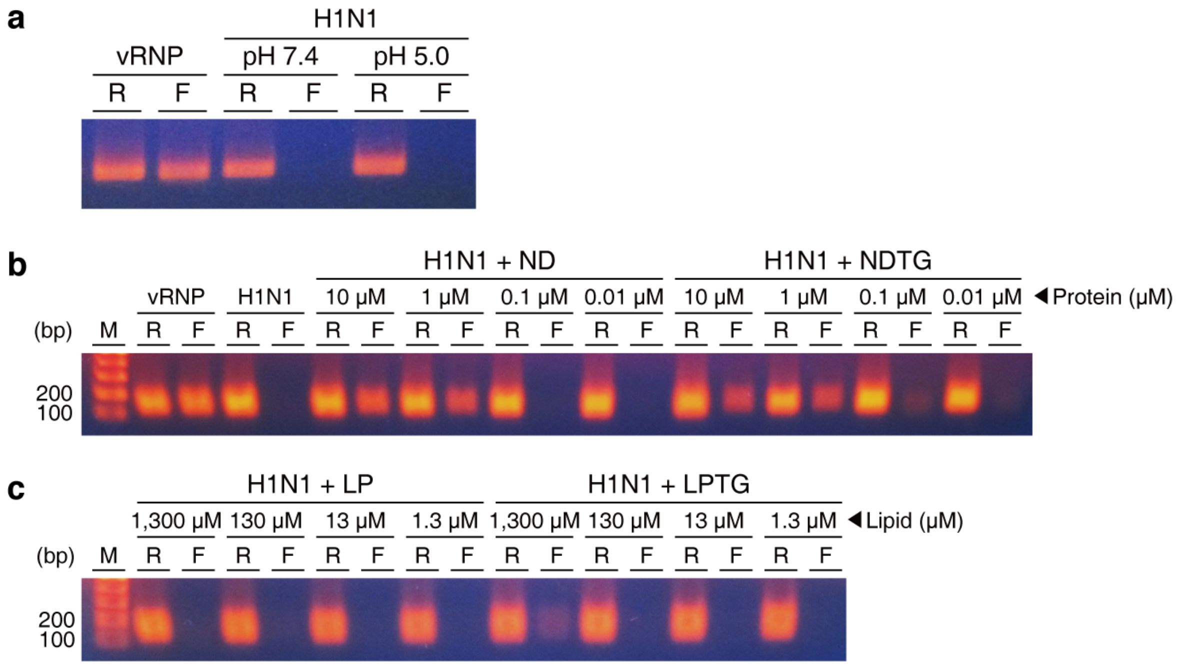

Supplementary Figure 5 | vRNP release from viruses by nanodiscs. Viruses (10 µM viral lipid) mixed with nanodiscs (0.01, 0.1, 1, or 10 µM protein) or liposomes (1.3, 13, 130, or 1,300 µM lipids) were incubated at 37°C for 15 min with constant shaking for virus–nanodisc/liposome binding. After the pH was decreased from 7.4 to 5.0 with 0.1 M citric acid, the mixtures were further incubated at 37°C for 20 min. A centrifugal filter with a 100-kDa molecular weight cutoff was used to separate the released vRNPs from intact viruses. After centrifugal filtration of the fusion products of H1N1 virus and NDTG at pH 5, vRNPs in the filtrates were analysed by RT-PCR to detect the viral M gene. **a**, Gel image showing that the centrifugal filter (100-kDa molecular weight cutoff) allowed vRNPs to pass through, but not intact virus at both pH 7.4 and 5.0. (**b** and **c**) vRNPs were released from viruses when the viral envelope fused with nanodiscs while virus–liposome fusion did not induce vRNP release. The samples derived from the same experiment and gels were processed in parallel (**b** and **c**). R, retentate; F, filtrate.

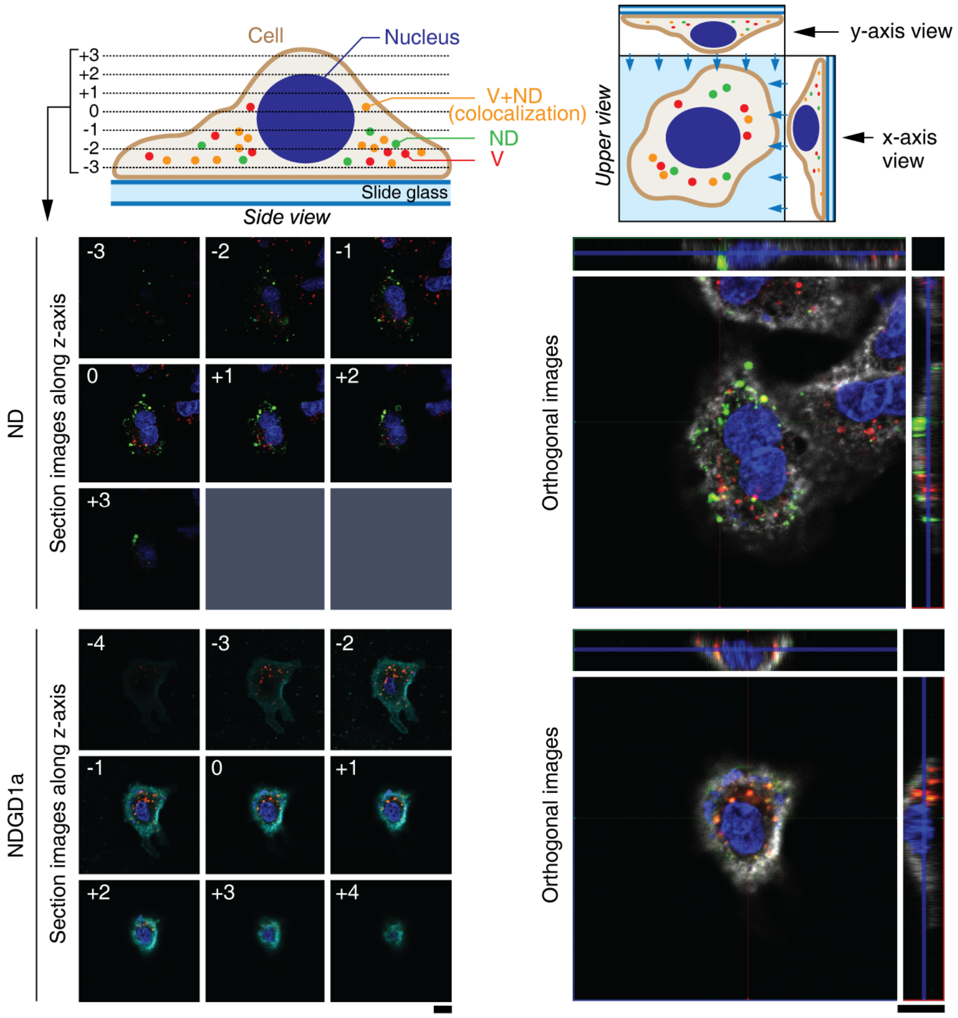

Supplementary Figure 6 | Analysis of localization of nanodiscs and virus in A549 cells from orthogonally-viewed confocal images. Immunofluorescent confocal images for intracellular colocalization of virus–nanodisc in a receptor-dependent manner were investigated. A549 cells were treated with SP-DiOC18-labeled influenza virus A/PR/8/34 (red) pre-mixed with Rhod-PE-labeled nanodiscs (green) at 37°C, and subsequently fixed at 2 h post-infection. Cell membranes and nuclei were stained with WGA-AF647 (white) and Hoechst (blue), respectively. Shown are z-stack images numbered for each section obtained along the z-axis with an image taken at the mid-height of the nucleus defined as zero (left), and orthogonal images viewed on x- and y-axis (right). Orange spots indicate colocalization. Scale bars, 10 μm.

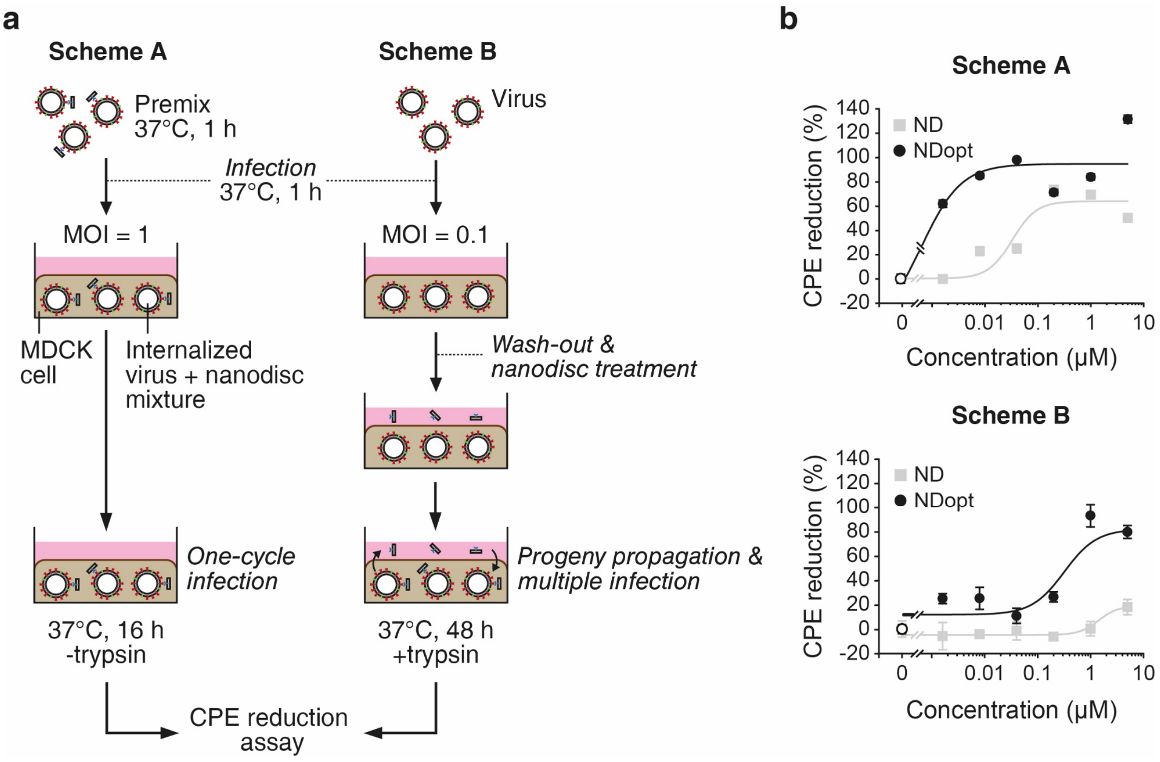

Supplementary Figure 7 | The effects of addition time of nanodisc and virus on the antiviral activity. To evaluate the effect of addition time of nanodisc–virus on antiviral activity of NDopt, CPE reduction assays following two schemes were performed. **a**, (Scheme A) A direct contact between nanodiscs and virus was made *via* pre-incubation of viruses and nanodiscs at 37°C for 1 h. CPE reduction during a single viral replication (37°C, 16 h) was measured in the absence of trypsin. (Scheme B) A direct contact between nanodiscs and virus was avoided by treating pre-infected cells with nanodiscs. After internalization of viruses at 37°C for 1 h the infected cells were treated with nanodiscs after washing out viruses in the medium. CPE reduction at the post-infection time 24 or 48 h was measured. For multiple cycles of viral replication, TPCK-treated trypsin was supplemented in the medium to activate hemagglutinin. **b**, CPE reduction was insignificant at 24 h post-infection. Scheme B yielded a relatively weak but dose-dependent antiviral effect of nanodisc at 48 h post-infection, suggesting that newly produced viral progenies were also neutralized by the nanodisc present in the medium, perhaps following the virus–nanodisc mixture state shown in Scheme A.

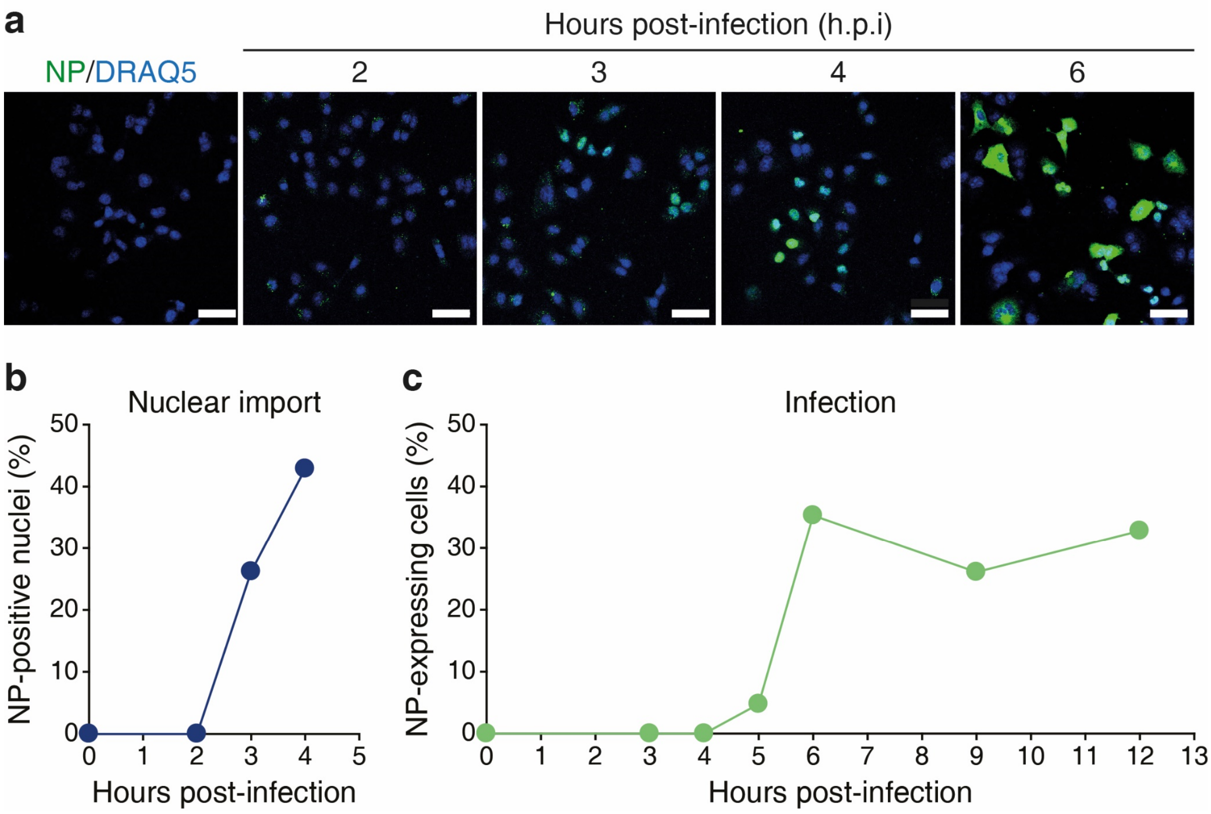

Supplementary Figure 8 | Detection of a single cycle of influenza virus replication by confocal microscopy. To investigate the viral life cycle, A549 cells were infected with A/PR/8/34 H1N1 virus and incubated at 37°C for the indicated time periods. After fixation, the cells were permeabilised with 0.5% Triton X-100, treated with primary mouse anti-NP antibodies, and incubated with secondary goat anti-mouse AF488 antibodies. Nuclei were stained with DRAQ5 (blue). **a**, vRNP nuclear import and newly synthesized NP became apparent over time (green). Scale bars, 50 µm. Nuclear import of vRNP, **b**, and translation of NP in the cells, **c,** were calculated.

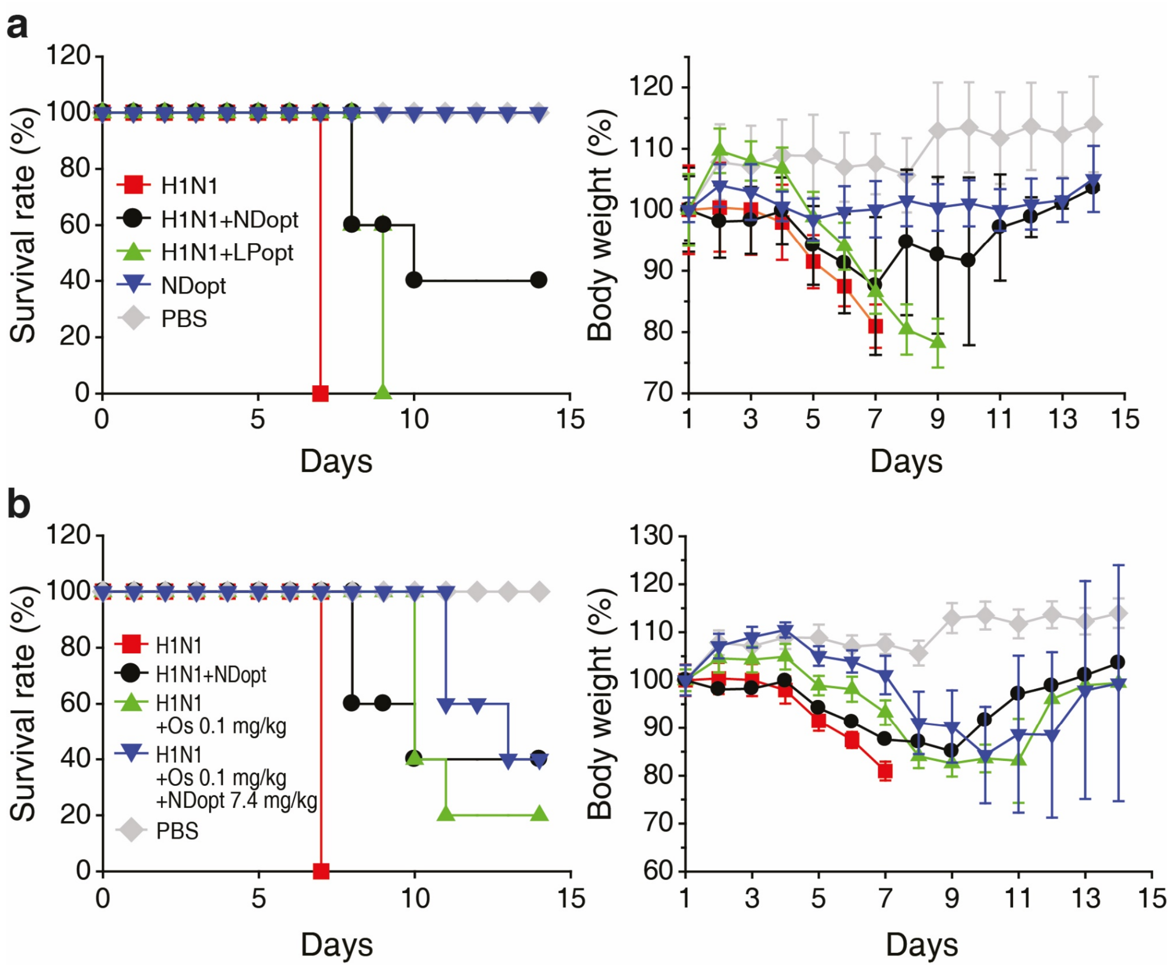

Supplementary Figure 9 | *In vivo* activity of NDopt. a, NDopt (7.4 mg/kg) and LPopt (7.4 mg/kg) were intraperitoneally administered to 6-week-old Balb/c mice (*n* = 5/group) at 1 h after intranasal challenge with two-fold 90% lethal doses (LD_{90}) of H1N1 virus, and then twice per day for 6 days beginning on the day of infection. The survival rate (left) and body weight (right) of each group were monitored daily for 14 days. Data are expressed as the mean ± s.d. **b**, Co-administration of NDopt and Os *in vivo*. NDopt (7.4 mg/kg) and Os (0.1 mg/kg) were intraperitoneally co-administered to 6-week-old Balb/c mice at 1 h after virus infection and then twice per day for 6 days beginning on the day of infection. NDopt (7.4 mg/kg, black) and Os (0.1 mg/kg, green) were used as monotherapy controls, respectively. Survival rate (left) and body weight (right) were monitored daily for 14 days. Data are expressed as the mean ± s.d.

Supplementary Note

Supplementary Note 1. Probability of intact progeny formation in a multiple infected cell when one or more vRNPs are trapped in the endosome by nanodisc.

We calculated the probability that 8 vRNPs are released into the cytosol when at least one vRNP per virus is trapped inside endosome and two or more viruses simultaneously enter the same cell.

(1) The actual number of viruses that can infect a particular cell and the probability of infection can be calculated by the Poisson distribution described by Ellis and Delbrück[4].

$P(n) = m^n e^{-m} (n!)^{-1}$, where

m = the multiplicity of infection or MOI,

n = the number of viruses that enter the host cell,

P(n) = the probability that a host cell will get infected by n viruses.

Then, $P(n>0) = 1 – P(n=0) = 1 – e^{-m}$

When MOI of 1 (one viral particle per cell) is used to infect a population of cells, the probability that the cell will not get infected is P(0) = 0.37. The probability that the cell will be infected by a single virus, two viruses and three viruses are P(1) = 0.37, P(2) = 0.18, and P(3) = 0.06, respectively.

The sum of the probabilities that the cell is multiply infected is 0.37 at MOI 1.

(2) The probability that virus replicates after infecting the host cells at MOI 1.

The probability, Q(n), that virus replicates normally from the host cell infected by n viruses when one of eight vRNPs per virus is trapped in the endosome is as follows:

$Q(1) = 0$,

$Q(2) = \{1-(1/{}_8C_1)\} = 0.875$,

$Q(3) = \{1-(1/{}_8C_1)^2\} = 0.984$

where, nCr stands for number of different combinations of n distinct objects taken r at a time.

Considering both virus infection probability and replication probability,

$P(1)Q(1) = 0$

$P(2)Q(2) = 0.16$

$P(3)Q(3) = 0.06$

The reduction of probability of virus replication is ~78% when one of the eight vRNPs is trapped inside endosome.

(3) If 2 or more vRNPs per virus are trapped inside endosome the reduction of probability of virus replication is increased to above ~85%. Thus, inhibition of virus infection and replication by vRNP entrapment is dramatic even when insufficient vRNPs can be complemented by simultaneously infected viruses.

Supplementary References

1 Ellens, H., Bentz, J., Mason, D., Zhang, F. & White, J. M. Fusion of influenza hemagglutinin-expressing fibroblasts with glycophorin-bearing liposomes: role of hemagglutinin surface density. *Biochemistry* **29**, 9697–9707 (1990).

2 Bentz, J., Ellens, H. & Alford, D. An architecture for the fusion site of influenza hemagglutinin. *FEBS Lett.* **276**, 1–5 (1990).

3 Ivanovic, T., Choi, J. L., Whelan, S. P., van Oijen, A. M. & Harrison, S. C. Influenza-virus membrane fusion by cooperative fold-back of stochastically induced hemagglutinin intermediates. *eLife* **2**, e00333 (2013).

4 Ellis, E. L. & Delbruck, M. The growth of bacteriophage. *J Gen. Physiol.* **22**, 365–384 (1939).
